# Septic shock and meningitis caused by *Capnocytophaga canimorsus* (serovar B) in an immunocompetent patient: case report

**DOI:** 10.1128/asmcr.00076-24

**Published:** 2025-01-29

**Authors:** Zoja Germuskova, Marc Westerholt, Lukas Frans Ocias

**Affiliations:** 1 Institute of Medical Microbiology, University of Zurich, Zurich, Switzerland; 2 Department of Clinical Microbiology, Karlstad Hospital, Karlstad, Sweden; 3 Center for Clinical Research and Education, Region Värmland 4210 , Karlstad, Sweden; 4 Department of Biomedical and Clinical Sciences, Linköping University 4566 , Linköping, Sweden; 5 Department of Laboratory Medicine, Clinical Microbiology, Faculty of Medicine and Health, Örebro University, Örebro, Sweden; Vanderbilt University Medical Center, Nashville, Tennessee, USA

**Keywords:** *Capnocytophaga canimorsus*, meningitis, immunocompetent, case, serovar

## Abstract

**Background:**

*Capnocytophaga canimorsus*, a Gram-negative rod found in the commensal oral microflora of dogs, is a rare cause of infection following a dog bite, with severe infections more commonly occurring in asplenic or immunocompromised patients. This is a rare case of septic shock and meningitis with a serovar B strain in an immunocompetent patient who passed away due to the infection.

**Case Summary:**

A previously healthy man in his late 50s with no known immunodeficiencies was admitted to the hospital with fever and cognitive deficits after being bitten by a dog. *Capnocytophaga canimorsus* grew in his cerebrospinal fluid and blood cultures. Despite aggressive fluid therapy, antibiotics, and intensive care, the patient succumbed to the infection. The strain was later characterized as serovar B using whole genome sequencing.

**Conclusion:**

Even immunocompetent patients are at risk of severe infection caused by *Capnocytophaga canimorsus*. These infections are an important differential diagnosis in patients presenting with fever following recent dog exposure with serovars A–C being the most common in human clinical isolates.

## INTRODUCTION

*Capnocytophaga canimorsus* (*C. canimorsus*) is a fermenting Gram-negative rod belonging to the family *Flavobacteriaceae*. The genus *Capnocytophaga* consists of several capnophilic species, which constitute a part of the commensal oral microflora in humans and domestic animals like dogs and less frequently cats (1).

The incidence of bloodstream infections has been reported to range between 0.5 and 4.1 cases per million inhabitants per year (2, 3), and for meningitis 0.03 cases per million inhabitants per year (4).

Several risk factors for *C. canimorsus* meningitis have been reported, including male gender, animal exposure, alcohol overconsumption, and immunosuppression (4, 5). A mortality of 3%–4% has been reported in patients with *C. canimorsus* meningitis, which is significantly lower than in patients with isolated bloodstream infections (4, 5). In addition, patients with meningitis have a significantly higher median time from exposure to the start of symptoms (5).

The capsular polysaccharide structures of *C. canimorsus* strains are essential to the evasion of the innate immune system by impeding macrophages from performing phagocytosis and are a recognized virulence factor. Although there is a large diversity of capsular types in *C. canimorsus*, three of these serovars (A–C) account for 88% of human isolates independent of geographical area, suggesting they are more pathogenic in humans. Serovar B, specifically, was found 14.6-fold more frequently in human clinical isolates compared to isolates from animals (6).

Antimicrobial susceptibility testing has shown that most strains of *C. canimorsus* are susceptible to beta-lactams, chloramphenicol, clindamycin, erythromycin, fluoroquinolones, rifampicin, tetracyclines, and vancomycin. Resistance or reduced susceptibility to aminoglycosides, aztreonam, colistin, metronidazole, and trimethoprim-sulfamethoxazole has, however, been reported in the majority of strains (5). In this paper, we describe a case of severe sepsis and meningitis caused by *C. canimorsus* in an immunocompetent patient.

## CASE PRESENTATION

A man of caucasian descent in his late 50s with some alcohol overconsumption but no prior medical history and no known immunosuppression was admitted to the emergency ward of a regional hospital in Sweden. At the time of admission, he presented with fever and cognitive deficits (i.e., disoriented at the current time, personal information, and trouble following instructions), which led to an early tentative diagnosis of bacterial meningitis. Around a week prior to the onset of symptoms, the patient was bitten by a dog and several small wounds were observed on the body during clinical examination. The bite wounds did not appear to be clinically infected and no antimicrobial post-exposure prophylaxis was given. Initial vital parameters showed a pulse of 122 bpm, a blood pressure of 142/85 mmHg, a temperature of 39.2°C, a respiratory frequency of 20 per minute, and a saturation of 97%. Approximately 30 minutes later, blood pressure dropped to 94/60, and within the span of 3 hours, the patient developed circulatory and respiratory failure due to septic shock and was transferred to the intensive care unit where the patient was intubated, received vasopressors, and was given over 6 L of intravenous fluids. The patient remained hemodynamically unstable despite intensive care and succumbed to the infection in less than 12 hours following admission. Biochemical analyses are presented in Table 1.

**TABLE 1:** Biochemical analyses of blood and cerebrospinal fluid

Characteristic	Value
Blood	
C-reactive protein (<5 mg/L)	132 mg/L
Creatinine (60–105 µmol/L)	153 µmol/L
Bilirubin (<25 µmol/L)	27 µmol/L
Alaninaminotransferasis (ALAT) (0.15–1.1 µkat/L)	1.53 µkat/L
Thrombocytes (145–348 × 10^9^/L)	9 × 10^9^/L
INR (<1.25)	2.8
Lactate (<1.3 mmol/L)	8.3 mmol/L
Cerebrospinal fluid	
Leucocytes (<5 × 10^6^/L)	9 × 10^6^/L
Monocytes	7 × 10^6^/L
Polymorphonuclear neutrophils	2 × 10^6^/L
Lactate (1.2–2.1 mmol/L)	5.6 mmol/L
Glucose	3.5 mmol/L
Albumin (<420 mg/L)	521 mg/L
Erythrocytes	1,300 × 10^6^/L

A CT angiography of the head and neck did not show any acute hemorrhage or occlusions, but a thoracic X-ray revealed pulmonary congestion and consolidations on the left side.

A Biofire FilmArray Meningitis/Encephalitis Panel (BioFire Diagnostics, Salt Lake City, USA) was unable to detect any of the 14 pathogens included in the panel in the patient’s cerebrospinal fluid (CSF). Both blood and CSF were incubated on hematin-agar in a high CO_2_ environment from the anaerobic bottles and displayed growth of *C. canimorsus* on days 5 and 7, respectively, confirming the diagnosis of bacterial meningitis. Identification of the strain was performed using matrix-assisted laser desorption-ionization time of flight (MALDI-TOF) mass spectrometry with MALDI Biotyper (Bruker Daltonik, Bremen, Germany), and was congruent with the microscopic findings of Gram-negative rods observed in the blood. The CSF culture was incubated longer than usual due to the early identification of *C. canimorsus* on day 1 using MALDI-TOF directly on the anaerobic bottle.

Antimicrobial susceptibility testing of the strain was performed using gradient testing (E-test, BioMérieux, Marcy-lÈtoile, France) on hematin-agar under high CO_2_ conditions due to scarce growth on Mueller-Hinton Fastidious agar. Formal categorizing of the susceptibility of the organism was not possible, as no species-specific breakpoints had yet been established by the European Committee on Antimicrobial Susceptibility Testing (EUCAST). However, all minimal inhibitory concentration (MIC) values were below the pharmacokinetic/pharmacodynamic (PK/PD) breakpoints for aerobic Gram-negative organisms, suggesting susceptibility to all tested antibiotics. The following MICs were observed: ≤0.016mg/L for ampicillin (PK/PD breakpoint: 8mg/L), 0.016mg/L for cefotaxime (PK/PD breakpoint: 0.5mg/L), <0.002 for meropenem (PK/PD breakpoint: 2mg/L), and 0.004 for ciprofloxacin (PK/PD breakpoint: 0.25mg/L).

Due to the suspicion of bacterial meningitis, the patient was initially treated empirically with a single dose of 3 g cefotaxime and 3 g ampicillin. A single dose of 750 mg aciclovir was administered after 4 hours and a single dose of 2 g meropenem was administered 8 hours after admission. In addition, the patient received a single dose of 500 mg levofloxacin and 100 mg hydrocortisone within the final hour.

In order to determine the capsular serovar, the isolate underwent whole genome sequencing (Illumina), and the species was confirmed as *C. canimorsus* (alignment identity of 98.5% with *C. canimorsus* strain ATCC 35979 16S ribosomal RNA). Subsequently, *in silico* PCR was performed on the assembled sequence using a PCR-reaction simulation script with published primer sequences for capsular serotyping by PCR (6, 7). Based on this analysis, the strain was classified as serovar B.

Additionally, the strain was analyzed for the presence of antimicrobial resistance genes (ABRicate with the NCBI AMRFinderPlus database) and possessed no known resistance genes in accordance with the phenotypic antimicrobial susceptibility testing (8).

## DISCUSSION

The patient presented a rare case of septic shock with concomitant meningitis caused by *C. canimorsus* in a male with no prior medical history and no known immunosuppression. Despite reporting some alcohol consumption, the magnitude of the patient’s alcohol consumption was not quantified in the medical records. *C. canimorsus* meningitis has a significantly lower mortality rate than *C. canimorsus* sepsis (4, 5). A possible explanation is that patients who develop meningitis may be less prone to develop a fatal infection, thus surviving the initial phase of the infection and granting the bacteria time to spread to the CNS (5, 9). The majority of patients with *C. canimorsus* infections are male, possibly due to a higher likelihood of males undergoing splenectomy due to motor vehicle accidents and alcoholism occurring twice as often in the male population (9). A recent review suggests that sepsis is the most frequent clinical presentation in immunocompetent patients with *C. canimorsus* infection, followed by meningitis, abdominal infections, and endocarditis or aortitis (10). Few case reports have included the serotype of the *C. canimorsus* strain, but these will likely increase as sequencing and bioinformatics gain more widespread adoption. In a case report similar to ours but without meningitis, an immunocompetent woman in her mid-50s developed fatal septic shock with Waterhouse-Friderichsen syndrome caused by a serovar B strain of *C. canimorsus* (11). In a Letter to the Editor by Vecilla et al., a case describing an immunocompetent 50-year-old male with fever caused by a serovar B strain of *C. canimorsus* is presented. The patient recovered following antibiotic treatment with meropenem (12).

This case highlights *C. canimorsus* infection as an important differential diagnosis in patients presenting with fever or signs of meningitis following recent dog exposure, even in immunocompetent individuals. Several capsular serovars have been implicated in human infection, the majority of which belong to serovars A, B, and C.

## Data Availability

The data from this study, including the genetic sequence of the isolate, have been deposited in the European Nucleotide Archive (ENA) at EMBL-EBI under accession number PRJEB83582.

## References

[1] Gaastra W, Lipman LJA. 2010. Capnocytophaga canimorsus. Vet Microbiol 140:339–346. doi:10.1016/j.vetmic.2009.01.040 19268498

[2] van Dam AP, Jansz A. 2011. Capnocytophaga canimorsus infections in the Netherlands: a nationwide survey. Clin Microbiol Infect 17:312–315. doi:10.1111/j.1469-0691.2010.03195.x 20167010

[3] Hästbacka J, Hynninen M, Kolho E. 2016. Capnocytophaga canimorsus bacteremia: clinical features and outcomes from a Helsinki ICU cohort. Acta Anaesthesiol Scand 60:1437–1443. doi:10.1111/aas.12752 27251795

[4] van Samkar A, Brouwer MC, Schultsz C, van der Ende A, van de Beek D. 2016. Capnocytophaga canimorsus meningitis: three cases and a review of the literature. Zoonoses Public Health 63:442–448. doi:10.1111/zph.12248 26693951

[5] Butler T. 2015. Capnocytophaga canimorsus: an emerging cause of sepsis, meningitis, and post-splenectomy infection after dog bites. Eur J Clin Microbiol Infect Dis 34:1271–1280. doi:10.1007/s10096-015-2360-7 25828064

[6] Hess E, Renzi F, Koudad D, Dol M, Cornelis GR. 2017. Identification of virulent Capnocytophaga canimorsus isolates by capsular typing. J Clin Microbiol 55:1902–1914. doi:10.1128/JCM.00249-17 28381610 PMC5442547

[7] Ozer EA. 2017. In-silico-Pcr: Github Repository. Available from: https://github:com/egonozer/in_silico_pcr

[8] Abricate TS. 2020. Github Repository. Available from: https://github:com/tseemann/abricate

[9] Prasil P, Ryskova L, Plisek S, Bostik P. 2020. A rare case of purulent meningitis caused by Capnocytophaga canimorsus in the czech republic - case report and review of the literature. BMC Infect Dis 20:100. doi:10.1186/s12879-020-4760-2 32013874 PMC6998360

[10] Mader N, Lührs F, Langenbeck M, Herget-Rosenthal S. 2020. Capnocytophaga canimorsus – a potent pathogen in immunocompetent humans – systematic review and retrospective observational study of case reports . Infect Dis (Lond) 52:65–74. doi:10.1080/23744235.2019.1687933 31709860

[11] Fernández Vecilla D, Angulo López I, Calvo Muro FE, Aspichueta Vivanco C, Renzi F, Pereda Martínez ME, Rosselló Soria J, Díaz de Tuesta Del Arco JL. 2023. Fatal septic shock and waterhouse-friderichsen syndrome caused by serovar B Capnocytophaga canimorsus in an immunocompetent patient. case report and review. Rev Esp Quimioter 36:92–96. doi:10.37201/req/060.2022 36424730 PMC9910678

[12] Fernández Vecilla D, Calvo Muro FE, Renzi F, Díaz de Tuesta Del Arco JL. 2022. Sepsis caused by Capnocytophaga canimorsus in an immunocompetent patient. Rev Esp Quimioter 35:304–306. doi:10.37201/req/006.2022 35468716 PMC9134879

